# 

**Published:** 1879-06

**Authors:** 


### THE CONVENTION OF NEW ENGLAND DENTAL SOCIETIES

Will be held on June 5th and 6th, at Wesleyan Hall, No. 36 Bromfield Street. Boston, Mass.

Fifteeen essays are already secured from able members, thus insuring a very interesting and profitable meeting.

A full programme of Essayists and Subjects will be issued when completed.

CENTRAL COMMITTEE OF ARRANGEMENTS.

C. H. Osgood, J. H. Kidder, R. R. Andrews, H. C. Merriam, A. M. Dudley, Massachusetts Dental Society.

L. Goddard, Maine Dental Society.

C. W. Clement, New Hampshire Dental Society.

Jas. Lewis, Vermont Dental Society.

Geo. L. Pamele, Connecticut Dental Society.

W. P. Church, Rhode Island Dental Society.

C. A. Brackett, Conn. Valley Dental Society.

L. D. Shepard, Merrimac Valley Dental Association.

E. P. Bradbury, American Academy Dental Science,

J. H. Batchelder, Boston Society for Dental Improvement

C. H. Osgood, Chairman. A. M. Dudley, Salem, Secretary.

### THE NATIONAL DENTAL ASSOCIATION.

The eleventh annual meeting of this Association (late "Southern") will be held in Augusta, Georgia, commencing on the second Tuesday in July, 1879, at ten a. m. The profession is cordially invited to attend.

L. D. Carpenter, Cor. Sec., Atlanta, Ga.,

E. S. Chisolm, Rec. Sec., Tuscaloosa, Ga.

### HO! FOR NIAGARA.

Commutation rates will be given by one or more railroads to those who go from or through Cincinnati to attend the meeting of the American Dental Association, to be held on the first Tuesday of August, at Niagara Falls. The July Register will indicate the exact date and the road or roads.

### MEETING.

The annual meeting of the Kentucky State Dental Society will be held in Louisville, beginning on Tuesday, June 3rd, at ten o'clock a. m.

The following subjects are proposed for papers and discussions, viz.:

Do pulps of extracted teeth unite in replanting?

What are the conditions of the alveolar process, when there is abscess on the tooth.

The provisions of nature for the protection of exposed pulp.

Secondary dentine, Osseous pulps, exostosis, action and effects of dead teeth.

The different agents that produce disintegration of enamel and dentine, and their mode of action.

Action of remedial agents upon diseased conditions that cause absorption of the alveolar process and gums; vitiated conditions of the oral secretion resulting from mercurial taint.

The subjects of histology, operative and mechanical dentistry, by essays and discussions. All are cordially invited.

The State Board of Examiners will be in session during the meeting of the association, to receive applicants for certificates to practice dentistry, in accordance with the dental law of Kentucky.

All interested should avail themselves of this opportunity of complying with the requirements of the law; all such should bear in mind that after this meeting, if they have not conformed to it, they will be constantly amenable to its penalties, and furthermore, can not legally collect any bills for professional services.

*THE DENTAL REGISTER,*

A MONTHLY MAGAZINE DEVOTED TO THE INTERESTS OF

THE DENTAL PROFESSION.

**Terms Reduced to $2 50 per Year. Single Copies 25c.**

*EDITED BY PROF. J. TAFT, D. D. S.,*

AND PUBLISHED BY

SPENCER & CROCKER, Ohio Dental Depot.

The volume commences in January and closes in December.

The REGISTER being issued monthly is always fresh, therefore Indispensable to the practitioner who desires to keep posted up with the new inventions and improvements which are daily developed. Neither expense nor effort will be spared to give value and variety to its contents. Articles will be illustrated with well executed engravings whenever they will add to the interest or assist to explanation.

Its extensive circulation and frequency of issue make it one of the BEST DENTAL ADVERTISING MEDIUMS in the United States.

All subscrptions must begin with January or July.

Local or special notices, five lines of less, will be inserted for $3.00 each insertion; over five lines, 50 cts. per line.

All advertising bills payable in advance, or at furthest, after the issue of the first number containing the advertisement. All transient advertisements to be paid for in advance.

☞CONTRIBUTIONS SOLICITED.

Exclusively Editorial Communications should be addressed to Editor.

The latest number of the REGISTER may be ordered with or without other goods at any time, and all letters should be addressed to

**SPENCER & CROCKER, Ohio Dental Depot, Cincinnati, O.**

